# Embryonic manipulations modulate differential expressions of heat shock protein, fatty acid metabolism, and antioxidant-related genes in the liver of heat-stressed broilers

**DOI:** 10.1371/journal.pone.0269748

**Published:** 2022-07-15

**Authors:** Chris Major Ncho, Akshat Goel, Vaishali Gupta, Chae-Mi Jeong, Yang-Ho Choi

**Affiliations:** 1 Department of Animal Science, Gyeongsang National University, Jinju, Republic of Korea; 2 Division of Applied Life Sciences (BK21 Plus Program), Gyeongsang National University, Jinju, Republic of Korea; 3 Institute of Agriculture and Life Sciences, Gyeongsang National University, Jinju, Republic of Korea; Tokat Gaziosmanpasa Universitesi, TURKEY

## Abstract

In this study, the effects of in ovo feeding of γ-aminobutyric acid (GABA) and embryonic thermal manipulation (TM) on plasma biochemical parameters, organ weights, and hepatic gene expression in broilers exposed to cyclic heat stress (32 ± 1°C for 8 days) (HS) were investigated. A total of 175 chicks were assigned to five treatments: chicks hatched from control eggs (CON); chicks hatched from control eggs but exposed to HS (CON+HS); chicks hatched from eggs injected at 17.5 days of incubation with 0.6mL of 10% GABA and exposed to HS (G10+HS); chicks hatched from thermally manipulated eggs (39.6°C, 6h/d from embryonic days 10 to 18) and exposed to HS (TM+HS); chicks hatched from eggs that received both previous treatments during incubation and exposed to HS (G10+TM+HS). Results revealed that on day 36 post-hatch, hepatic NADPH oxidase 1 (P = 0.034) and 4 (P = 0.021) genes were downregulated in the TM+HS and G10+TM+HS compared to the CON+HS group. In addition, while acetyl-CoA carboxylase gene expression was reduced (P = 0.002) in the G10+TM group, gene expression of extracellular fatty acid-binding protein and peroxisome proliferator-activated receptor-γ was lower (P = 0.045) in the TM+HS group than in the CON+HS group. HS led to higher gene expression of heat shock protein 70 (HSP70) and 90 (HSP90) (P = 0.005, and P = 0.022). On the other hand, the TM+HS group exhibited lower expression of both HSP70 (P = 0.031) and HSP90 (P = 0.043) whereas the G10+TM+HS group had a reduced (P = 0.016) HSP90 expression compared to the CON+HS. MANOVA on different gene sets highlighted an overall lower (P = 0.034) oxidative stress and lower (P = 0.035) heat shock protein expression in the G10+TM+HS group compared to the CON+HS group. Taken together, the current results suggest that the combination of in ovo feeding of GABA with TM can modulate HSPs and antioxidant-related gene expression in heat-stressed broilers.

## Introduction

In recent years, poultry production has increased to such an extent that countries with tropical climates have become one of the major contributors to the industry. However, most of the birds’ genetic strains used have been selected under mild climates [1]. For meat-type chickens, rapid growth and muscle yield have been the main parameters for selection. Thus, as the muscle contributes to energy production, a larger portion of the muscle in their body makes them more sensitive to high rearing temperatures. Heat stress (HS) can result from exposure to excessively high ambient temperatures. In broilers, HS is known for its adverse effects on production performances, reproductive profile, and immune response [2–4].

Broiler’s performance during HS may be further impaired by the overproduction of reactive oxygen species (ROS) leading to oxidative stress [5]. Although ROS production is an essential component of aerobic cell respiration, the increase in ROS generation happening during HS might cause lipid peroxidation and cell apoptosis [6]. HS-related oxidative stress is usually accompanied by increased activity of enzymes such as NADPH oxidase (NOX) which are closely linked to ROS production. In poultry, the main antioxidant enzymes are superoxide dismutase (SOD), glutathione peroxidase (GPX), and catalase (CAT) [7]. This array of enzymes, triggered by the activation of the nuclear factor (erythroid-derived 2)-like 2 (NRF2), maintains redox homeostasis and prevents oxidative injury [7].

Aside from oxidative damage, hyperthermia in broilers has also been associated with disruptions in lipid metabolism [8]. Lipids are responsible for biological functions such as immunity, energy storage, and cell protection [9]. Evidence suggests that oxidative and HS interacting effects impair hepatic fatty acid synthesis in the avian liver [8]. In addition, chronic HS increased the proportion of abdominal fat in broilers, thus reducing economic profits [10]. As lipid peroxidation can lead to higher ROS production, HS mitigations strategies that can reduce lipid accumulation may alleviate heat-induced oxidative stress.

Several strategies have been tested to reduce the deleterious effects of HS in broilers. Among them is thermal acclimatization during incubation. Embryonic thermal manipulation (TM) consists of controlled exposure of the embryo to intermittent high temperatures during the incubation period. TM can provide long-lasting thermotolerance by mainly affecting metabolic processes and stress-related pathways due to epigenetic modifications [11]. Even though TM has been proven effective in helping broilers cope with HS, there is not yet a standardized protocol for its application. Although different time points and durations of TM are being actively tested, TM appeared to result in better HS adaptation when applied during the development of the hypothalamus-hypophysis-thyroid axis or -adrenal axis, or both [12]. Thus, the preponderance of studies has selected 7 to 18 days of incubation as the period for TM [13–15]. Our recent study found that increasing the incubation temperature to 39.6°C for 6 h daily between days 10 to 18 strongly modulated stress-related gene expression in hatchlings [16]. Other studies similar to ours have also reported reduced body core temperature, improved antioxidant status, and enhanced thermal resistance in broilers exposed to various HS conditions [17–19].

Another solution is in ovo feeding. In ovo feeding of substances such as prebiotics, could improve HS resistance of broilers [20, 21]. Our previous study also showed that in ovo feeding of γ-aminobutyric acid (GABA) could lower the expression of fatty acid metabolism-related genes in heat-stressed birds [22]. GABA is one of the major inhibitory neurotransmitters that play an important role in appetite, immunity, and energy metabolism [23]. GABA has been provided as a feed additive or through drinking water to combat HS in broilers and laying hens [24, 25]. Another study using oral gavage of a GABA solution found lower rectal temperatures along with higher jejunum activities of antioxidant enzymes, and triiodothyronine in the blood of broilers exposed to HS [26].

Therefore, in this study, we hypothesized that combining TM and in ovo feeding of GABA may enhance the response of broilers to HS. To verify our hypothesis, we evaluated organ weights, plasma biochemical parameters, as well as hepatic antioxidant-related, fatty acid metabolism-related, and heat shock proteins gene expression in broilers exposed to cyclic HS.

## Materials and methods

All the experimental procedures for this study were approved by the Institutional Animal Care and Use Committee of Gyeongsang National University (GNU-200916-C0058).

### Incubation, in ovo feeding and thermal manipulation procedures

Three hundred hatching eggs were obtained from 37-week-old Indian River breeder hens housed at a local broiler breeder farm (Hapcheon, Korea). Following standard incubation conditions, the eggs were set for incubation in two identical incubators with a capacity of 190 eggs each (Maru 190, Rcom Co., Ltd., Gimhae, Korea). One incubator was kept at standard incubation conditions until hatch while the other was used to perform TM from embryonic days (EDs) 10 to 18. Briefly, from ED 1 to 18, eggs were submitted to 37.8°C and 56% relative humidity (RH), and from ED 18 until hatch, the incubation temperature was maintained at 36.8°C with 70% RH. On ED 10, after candling, the non-fertilized eggs were removed from the incubators. The eggs were distributed in five groups of equal numbers (n = 48) with similar weights (61.0 ± 2g each group) using the Solver module of Microsoft Excel (Microsoft Excel 2016; Microsoft Corp., Redmond, WA, USA). Each group was composed of 8 replicates of 6 eggs. Because there were no significant differences in hatching and biological parameters between the distilled water-injected control and the non-injected control in our previous study [27], we included only the latter in this study. At ED 17.5, one group received in ovo injection of 0.6 mL of 10% GABA (#A2129, Sigma-Aldrich, Inc., St. Louis, MO, USA) dissolved in distilled water. The in ovo feeding procedure was the following: after disinfection with a 70% ethanol solution a small hole was drilled in the surface of the blunt end of the egg using a dental drill (Saeshin, Daegu, Korea). Thereafter, eggs were injected via the hole using a 1 mL syringe with a 23 G, 25 mm needle. The injection site targeted the amniotic sac of the embryo. For a second group, TM was performed by increasing incubational temperature to 39.6°C for 6 h daily from EDs 10 to 18. A third group received both in ovo injection of GABA and TM during incubation. Thus, only the second and third groups were set in the incubator in which TM was performed. Finally, the remaining two groups were incubated under the standard conditions aforementioned and were considered as controls. Our previous studies described both in ovo procedure and TM in more detail [27].

### Rearing conditions and HS challenge

**Fig 1:**
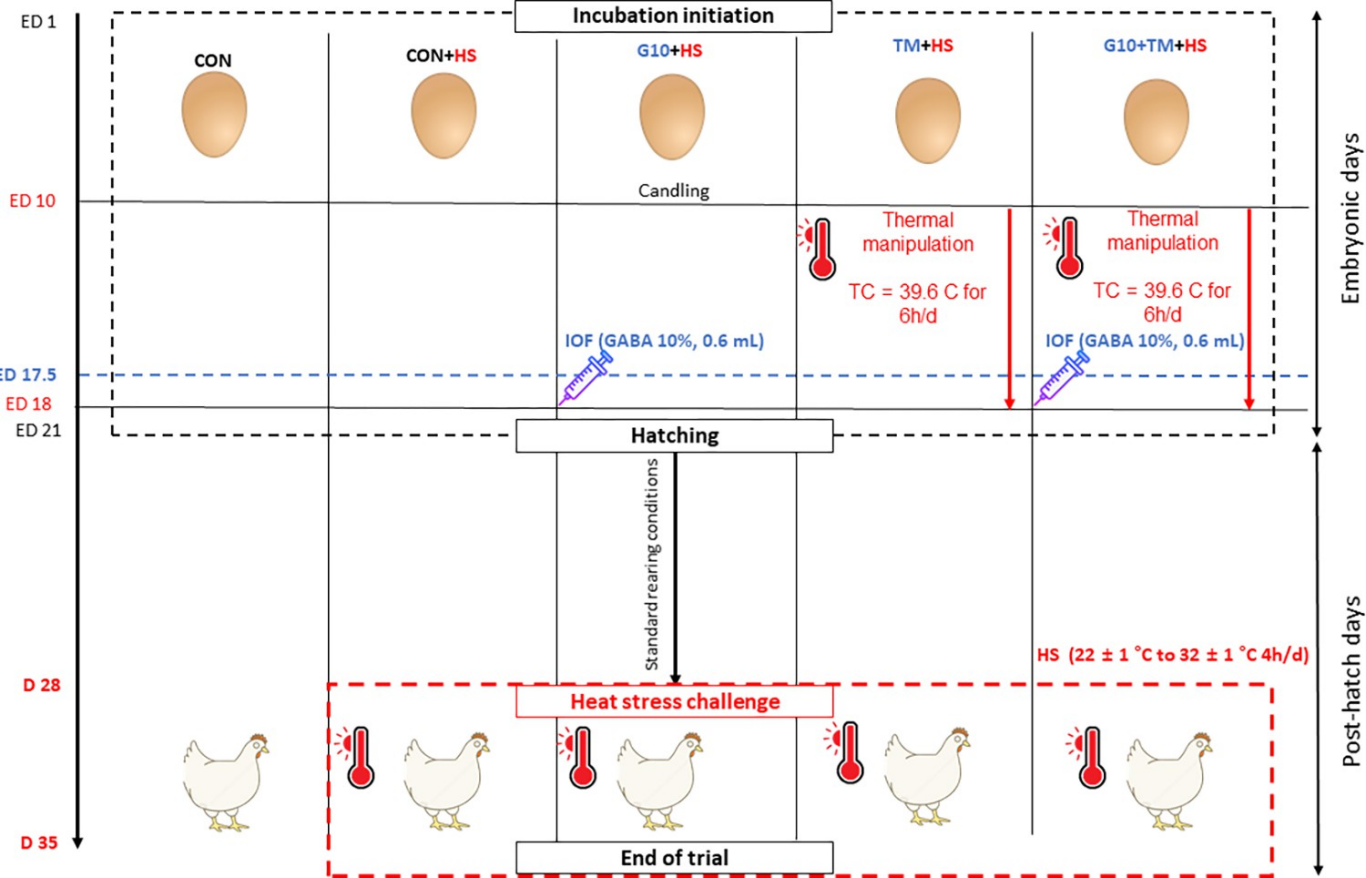
Study design. The treatments are described as follows: CON, chicks hatched from control eggs without in ovo injection and incubated at standard temperature; CON+HS, chicks hatched from control eggs without in ovo injection, incubated at standard temperature but exposed to HS; G10+HS, chicks hatched from eggs injected at 17.5 days of incubation with 0.6mL of 10% GABA dissolved in distilled water and exposed to HS; TM+HS, chicks hatched from thermally manipulated eggs exposed to 39.6°C for 6 h daily from ED 10 to 18 and exposed to HS; G10+TM+HS, chicks, hatched from eggs that received both previous treatments during incubation, and exposed to HS.

After hatching, a total of 175 unsexed one-day-old broiler chicks were raised in a room with 2 sets of H-type battery cage assemblies on each side of the room, 3 tiers on each side, and 7 cages per tier. The room had a thermally controlled environment of 34 ± 1°C and 50% relative humidity (RH), and then the temperature was gradually decreased to 22 ± 1°C on day 28. A commercially available feed (Nonghyup Feed Co., Seoul, Korea) in crumbled form and water were provided to the birds ad-libitum under 23 h of light and 1 h of dark. On day 28, the chicks were allocated into five different treatment groups: chicks hatched from control eggs without in ovo injection and incubated at standard temperature (CON); chicks hatched from control eggs without in ovo injection, incubated at standard temperature but exposed to HS (CON+HS); chicks hatched from eggs injected at 17.5 days of incubation with 0.6mL of 10% GABA dissolved in distilled water and exposed to HS (G10+HS); chicks hatched from thermally manipulated eggs exposed to 39.6°C for 6 h daily from ED 10 to 18 and exposed to HS (TM+HS); chicks hatched from eggs that received both previous treatments during incubation and exposed to HS (G10+TM+HS). Each treatment had 5 cages of 7 chicks. The dimensions of the cages were 90 cm x 70 cm x 45 cm in length, width, and height, respectively. Thus, the stocking density was equal to 900 cm^2^ per bird. The birds were challenged with a cyclic HS between 28 and 35 days of age following a previously executed protocol with minor modifications [28]. An overview of the design is presented in Fig 1. In the HS room, the ambient temperature was gradually increased from 22 ± 1°C to 32 ± 1°C over 30 min, then the temperature was maintained to 32 ± 1°C for 4 h, and finally returned to 22 ± 1°C over 30 min whereas the chicks under thermoneutral temperature remained at 22 ± 1°C until day 35.

### Blood, tissues sampling, and plasma biochemical parameters analysis

On day 36, 24 h after the end of the HS challenge, one bird from each cage (5 per treatment) was randomly selected and euthanized with carbon dioxide immediately before blood and tissue sampling. Blood was collected from heart punctures and transferred into heparinized vacuum containers (#367874, BD Co., Ltd., Franklin Lakes, NJ, USA). The blood samples were then centrifuged at 2,000x g for 10 min at 4°C and plasma was collected and then stored at -20°C for later analysis. Tissues for the liver, spleen, bursa, and heart were sampled and weighed. Absolute and relative organ weights were respectively recorded and calculated after sampling. Relative organ weight was calculated according to the following formula:

Relativeorganweight(%)=(Absoluteorganweight(g)/Bodyweight(g))*100

Liver samples were snap-frozen in liquid N_2_ and stored at -80°C for further analysis. Plasma metabolite concentrations were measured according to the manufacturer guide using a VetTest Chemistry Analyzer (IDEXX Co., Ltd., Westbrook, ME, USA) with a dry-slide technology.

### Real-time PCR for mRNA quantification

Total RNA extraction from the liver was performed using Trizol™ reagent (ThermoFisher Scientific, Inc., Waltham, MA, USA) following the manufacturer’s protocol. RNA concentrations and purities of the samples were confirmed by reading the optical density of each sample in a Nanodrop (ThermoFisher Scientific, Inc., Waltham, MA, USA). Subsequently, the reverse transcription reaction was conducted using a PrimeScript^TM^ first-strand cDNA synthesis kit (Takara, Tokyo, Japan) following the manufacturer’s guide. The cDNA synthesized was then used to perform real-time PCR using a StepOnePlus™ system (Applied Biosystems, Inc., Waltham, MA, USA) according to the following protocol: 10 min at 95°C followed by 40 cycles of 15 s at 95°C and 1 min at 60°C. Each reaction well was composed of 20 μL containing Power SYBR^TM^ green PCR master mix (ThermoFisher Scientific, Inc.), and a 10 pmol concentration of forward and reverse primer specific for each gene and cDNA. Information related to the primers is presented in Table 1. The geometric mean of both reference genes (β-actin and GAPDH) was used to calculate the expression levels of the target genes [29]. Relative expression was determined using the 2^−ΔΔct^ algorithm. To visualize the downregulation vs. upregulation of genes between treatments, the Log_2_ fold changes were graphed [20].

**Table 1:** Oligonucleotide primer sequences for RT-qPCR.

Gene	Sequence	Accession number	Reference
ACC	F: CACTTCGAGGCGAAAAAC	XM_015295697.2	[27]
	R: GGAGCAAATCCATGACCA		
CAT	F: ACCAAGTACTGCAAGGCGAA	NM_001031215.1	[27]
	R: TGAGGGTTCCTCTTCTGGCT		
EXFABP	F: GGAGGACCTTGCACATGA	NM_205422.1	This study
	R: GTGTAGTTCCGCTCCCTA		
FAS	F: CAATGGACTTCATGCCTC	NM_205155.3	[27]
	R: GCTGGGTACTGGAAGACA		
GPx1	F: AACCAATTCGGGCACCAG	NM_001277853.2	[27]
	R: CCGTTCACCTCGCACTTCTC		
HSP70	F: CCCGAGCAAGCTGGATTCT	AY143693.1	[27]
	R: CAGGAGCAGATCTTGCACATTT		
HSP90	F: GCGAAGACGTGTTCCTGTAT	NM_001109785	[22]
	R: GGTCATCCCTATGCCGGTATC		
NOX1	F: GCGAAGACGTGTTCCTGTAT	NM_001101830.1	[22]
	R: GAACCTGTACCAGATGGACTTC		
NOX4	F: CCTCTGTGCTTGTACTGTGTAG	NM_001101829.1	[22]
	R: GACATTGGAGGGATGGCTTAT		
NRF2	F: CAGAAGCTTTCCCGTTCATAG-A	NM_205117	This study
	R: GACATTGGAGGGATGGCTTAT		
PPAR.G	F: TCAGGTTTGGGCGAATGC	XM_040646063.1	This study
	R: CGCTCGCAGATCAGCAGA		
SOD	F: AGGGGGTCATCCACTTCC	NM_205064.1	[22]
	R: CCCATTTGTGTTGTCTCCAA		
β-actin	F: ACCGGACTGTTACCAACA	NM_205518.1	This study
	R: GACTGCTGCTGACACCTT		
GAPDH	F: TTGGCATTGTGGAGGGTCTTA	NM_204305.1	[27]
	R: GTGGACGCTGGGATGATGTT		

Abbreviations: ACC, acetyl-CoA carboxylase; CAT, catalase; EXFABP, extracellular fatty acid-binding protein; FAS, fatty acid synthase; GAPDH, glyceraldehyde-3-phosphate dehydrogenase; GPx1, HSP70, heat-shock protein 70; HSP90, heat shock protein 90; glutathione peroxidase 1; NOX1, nicotinamide adenine dinucleotide phosphate oxidase 1; NOX4, nicotinamide adenine dinucleotide phosphate oxidase 4; NRF2, nuclear factor erythroid 2-related factor 2; PPAR-γ, peroxisome proliferator-activated receptor gamma; SOD, superoxide dismutase.

### Statistical analysis

The Shapiro Wilk and Levene’s tests were used for assessing the normality of distribution and the equality of variances. All percentage records below 20% were transformed by arcsine square root. Data of the organ indexes, plasma biochemical parameters, and hepatic gene expression were analyzed via one-way ANOVA using the “*GLM procedure*” of the SAS software version 9. 4 (SAS Institute Inc., 2009). A Tukey posthoc test was performed following a significant P-value (P < 0. 05) to assess differences among means. Data in the text are given as the mean ± standard error of the mean (SEM). Planned contrasts were made for the following comparison using the “*contrast statement*” of the SAS software [30]: control group vs control heat-stressed group; GABA injected group vs control heat-stressed group; thermally manipulated group vs control heat-stressed group and the combination of both treatment vs control heat-stressed group. Because three main categories of genes were evaluated in this trial, we further evaluated treatment effects on sets of genes (antioxidant related, fatty acid metabolism-related, and heat shock proteins) by one-way multivariate analysis of variance (MANOVA) and multivariate planned contrast [31] using the “*manova statement*” of the SAS software [32]. Before applying MANOVA, multivariate normality was assessed, and homogeneity of the variance-covariance matrix was tested using Box’s M test. The Wilks’ Lambda P < 0.05 was considered for rejecting the null hypothesis. All the assumptions evaluated by the Shapiro Wilk, Levene, and Box’s M test were met, thus conducting the previous parametric tests was justified.

Finally, to detect the potential associations between blood biochemical parameters, heat-shock proteins, antioxidant, and fatty acid metabolism-related genes, Pearson correlations were calculated using the “*CORR procedure*” of the SAS software. Statistical significance was declared at P < 0.1 for the correlation test. The heatmap was generated using GraphPad Prism 8 (GraphPad Software, Inc., La Jolla, CA, USA).

## Results

Multivariate analysis of gene expression was conducted because all genes evaluated belonged to a predefined category. There are three categories: antioxidant-related genes (CAT, SOD, GPx1, NRF2, NOX1, and NOX4); heat-shock proteins (HSP70 and HSP90); and fatty acid metabolism-related genes (ACC, FAS, EXFABP, and PPAR-γ). Moreover, pairwise correlation analysis revealed that Pearson’s r values were between the recommended range (See S1 Fig), justifying the appropriateness of conducting MANOVA [33]. The results regarding planned contrast performed on gene expressions can be found in S1 Table.

### Effects of in ovo feeding of GABA and TM on organ weights and plasma biochemical parameters

No significant differences were found between groups for absolute weights of the spleen, heart, and bursa (Table 2). However, absolute liver weight was higher (P = 0.001) in the CON+HS group, but lower (P = 0.001) in the TM+HS and G10+TM+HS groups, compared with the CON group. As a result, relative liver weight was significantly higher (P = 0.015) in the CON+HS treatment while there were no significant differences between the remaining groups (G10+HS, TM+HS, G10+TM+HS) and the CON group. The relative weight of bursa was significantly different (P = 0.003) among treatments with the highest value in the TM+HS and the lowest in the G10+HS.

**Table 2:** Effects of in ovo feeding of GABA and embryonic thermal manipulation on absolute and relative organs weight in broiler chickens exposed to cyclic HS.

Parameters	CON	CON+HS	G10+HS	TM+HS	G10+TM+HS	P-value
Absolute weight (g)						
Liver	50.2±3.8^a^	74.4±4.5^b^	60.1±3.0^ab^	55.9±3.3^a^	59.1±3.2^a^	0.001
Spleen	2.2±0.2	2.1±0.2	1.9±0.1	2.0±0.1	2.2±0.3	0.877
Bursa	3.4±0.2	3.8±0.4	3.0±0.2	3.7±0.4	2.9±0.1	0.08
Heart	10.5±0.6	10.4±0.5	10.0±0.5	10.3±0.6	9.1±0.4	0.379
Relative weight (%)						
Liver	2.4±0.07^a^	3.9±0.60^b^	2.8±0.20^ab^	2.8±0.20^ab^	2.9±0.08^ab^	0.015
Spleen	0.10±0.01	0.11±0.01	0.09±0.01	0.10±0.01	0.11±0.01	0.797
Bursa	0.16±0.01^abc^	0.19±0.01^bc^	0.14±0.01^a^	0.20±0.02^c^	0.15±0.01^ab^	0.003
Heart	0.50±0.07	0.54±0.01	0.46±0.0	0.52±0.02	0.45±0.01	0.328

The treatments are described as follows: CON, chicks hatched from control eggs without in ovo injection and incubated at standard temperature; CON+HS, chicks hatched from control eggs without in ovo injection, incubated at standard temperature but exposed to HS; G10+HS, chicks hatched from eggs injected at 17.5 days of incubation with 0.6mL of 10% GABA dissolved in distilled water and exposed to HS; TM+HS, chicks hatched from thermally manipulated eggs exposed to 39.6°C for 6 h daily from ED 10 to 18 and exposed to HS; G10+TM+HS, chicks hatched from eggs that received both previous treatments during incubation and exposed to HS. Data show mean ± SEM (n = 5). The P-values reported in the table are from the ANOVA procedure. Means with different superscripts (a, b or c) in the same row indicate significant differences by the Tukey test (P < 0.05).

Table 3 shows plasma glucose, triglycerides, total protein, and cholesterol concentrations which were not significantly different among treatment groups.

**Table 3:** Effects of in ovo feeding of GABA and embryonic thermal manipulation on plasma biochemical parameters in broiler chickens exposed to cyclic HS.

Parameters	CON	CON+HS	G10+HS	TM+HS	G10+TM+HS	P-value
Glucose (mg dL^-1^)	300.4±28.3	285.2±8.2	299.2±18.5	285.2±4.3	311.2±16.9	0.802
Triglycerides (mg dL^-1^)	62.2±17.7	58.0±16.1	50.4±10.1	54.0±8.5	55.0±15.1	0.981
Total protein (g.dL^-1^)	3.0±0.2	3.3±0.1	3.4±0.2	3.3±0.1	3.4±0.4	0.751
Cholesterol (mg dL^-1^)	106.0±4.1	136.8±6.6	100.4±17.0	123.2±9.4	113.4±4.1	0.096

The treatments are described as follows: CON, chicks hatched from control eggs without in ovo injection and incubated at standard temperature; CON+HS, chicks hatched from control eggs without in ovo injection, incubated at standard temperature but exposed to HS; G10+HS, chicks hatched from eggs injected at 17.5 days of incubation with 0.6mL of 10% GABA dissolved in distilled water and exposed to HS; TM+HS, chicks hatched from thermally manipulated eggs exposed to 39.6°C for 6 h daily from ED 10 to 18 and exposed to HS; G10+TM+HS, chicks hatched from eggs that received both previous treatments during incubation and exposed to HS. Data show mean ± SEM (n = 5).

### Effects of in ovo feeding of GABA and TM on hepatic antioxidant related gene expression

**Fig 2:**
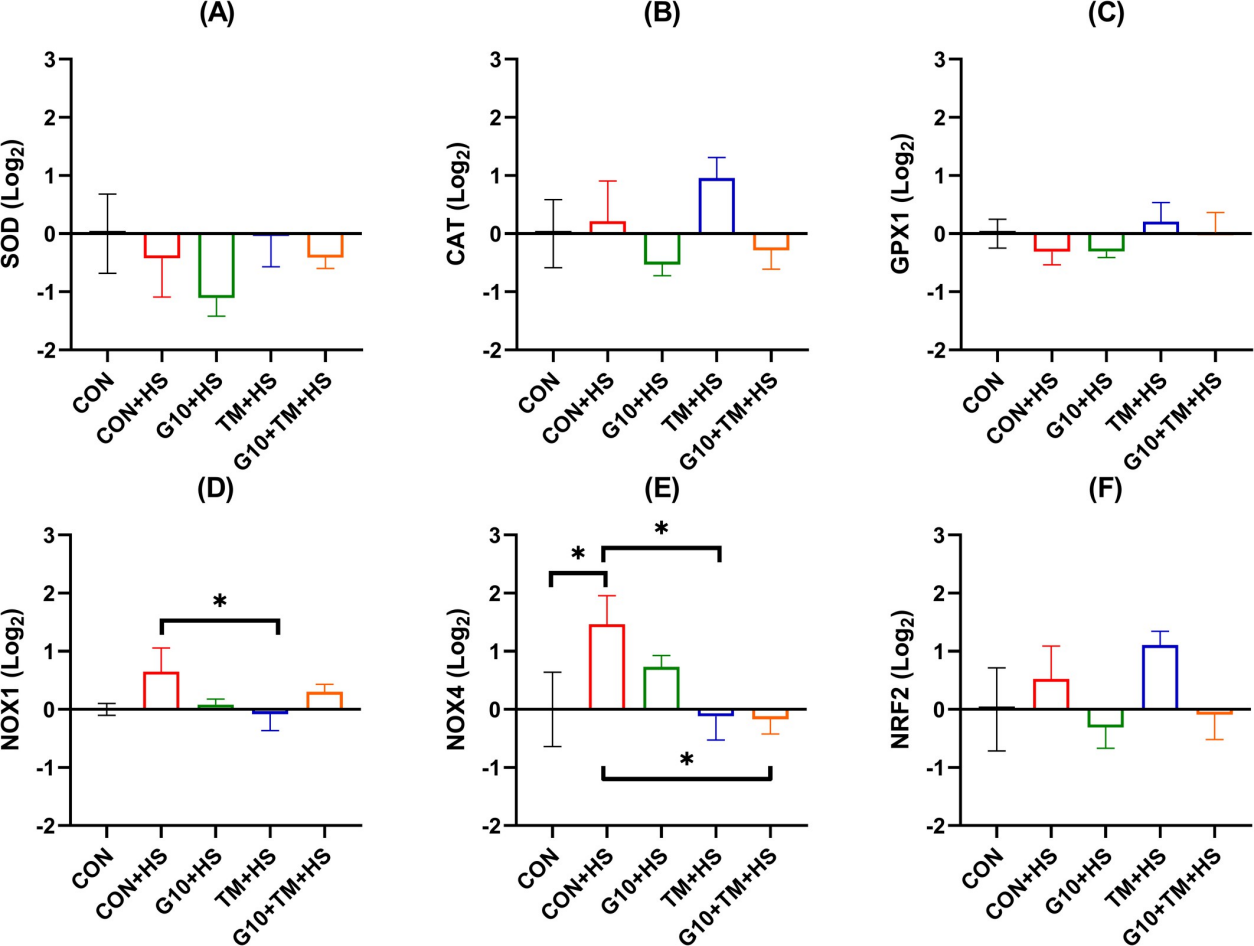
Effects of in ovo feeding of GABA and embryonic thermal manipulation on relative mRNA expression of hepatic SOD (A), CAT (B), GPx1 (C), NOX1 (D), NOX4 (E), and NRF2 (F) in broiler chickens exposed to cyclic HS. The treatments are described as follows: CON, chicks hatched from control eggs without in ovo injection and incubated at standard temperature; CON+HS, chicks hatched from control eggs without in ovo injection, incubated at standard temperature but exposed to HS; G10+HS, chicks hatched from eggs injected at 17.5 days of incubation with 0.6mL of 10% GABA dissolved in distilled water and exposed to HS; TM+HS, chicks hatched from thermally manipulated eggs exposed to 39.6°C for 6 h daily from ED 10 to 18 and exposed to HS; G10+TM+HS, chicks hatched from eggs that received both previous treatments during incubation and exposed to HS. Data show mean ± SEM (n = 5). Results of the contrast analysis are indicated in the graph. Abbreviations: CAT, catalase; GPx1, glutathione peroxidase 1; NOX1, nicotinamide adenine dinucleotide phosphate oxidase 1; NOX4, nicotinamide adenine dinucleotide phosphate oxidase 4; NRF2: Nuclear factor erythroid 2-related factor 2; SOD, superoxide dismutase.

Fig 2 shows the effects of GABA in ovo feeding and TM on the relative expression of antioxidant-related genes in the liver. TM and GABA did not influence the regulation of SOD, CAT, GPX1, and NRF2 genes.

On the other hand, the TM+HS group had a lower NOX1 expression compared with the CON+HS (P = 0.034). Higher NOX4 gene expression (P = 0.032) was observed in the CON+HS group compared to the CON group. The TM+HS and G10+TM+HS treatments resulted in a downregulation (P = 0.021 and P = 0.024) of the NOX4 gene expression when compared to the CON+HS group. Interestingly, there was an overall increase (P = 0.034, MANOVA) in the expression of antioxidant-related genes by HS (Fig 5A). In addition, the G10+TM+HS treatment led to downregulation (P = 0.003, MANOVA) of the same set of genes when compared to the CON+HS group.

### Effects of in ovo feeding of GABA and TM on hepatic fatty acid metabolism-related gene expression

**Fig 3:**
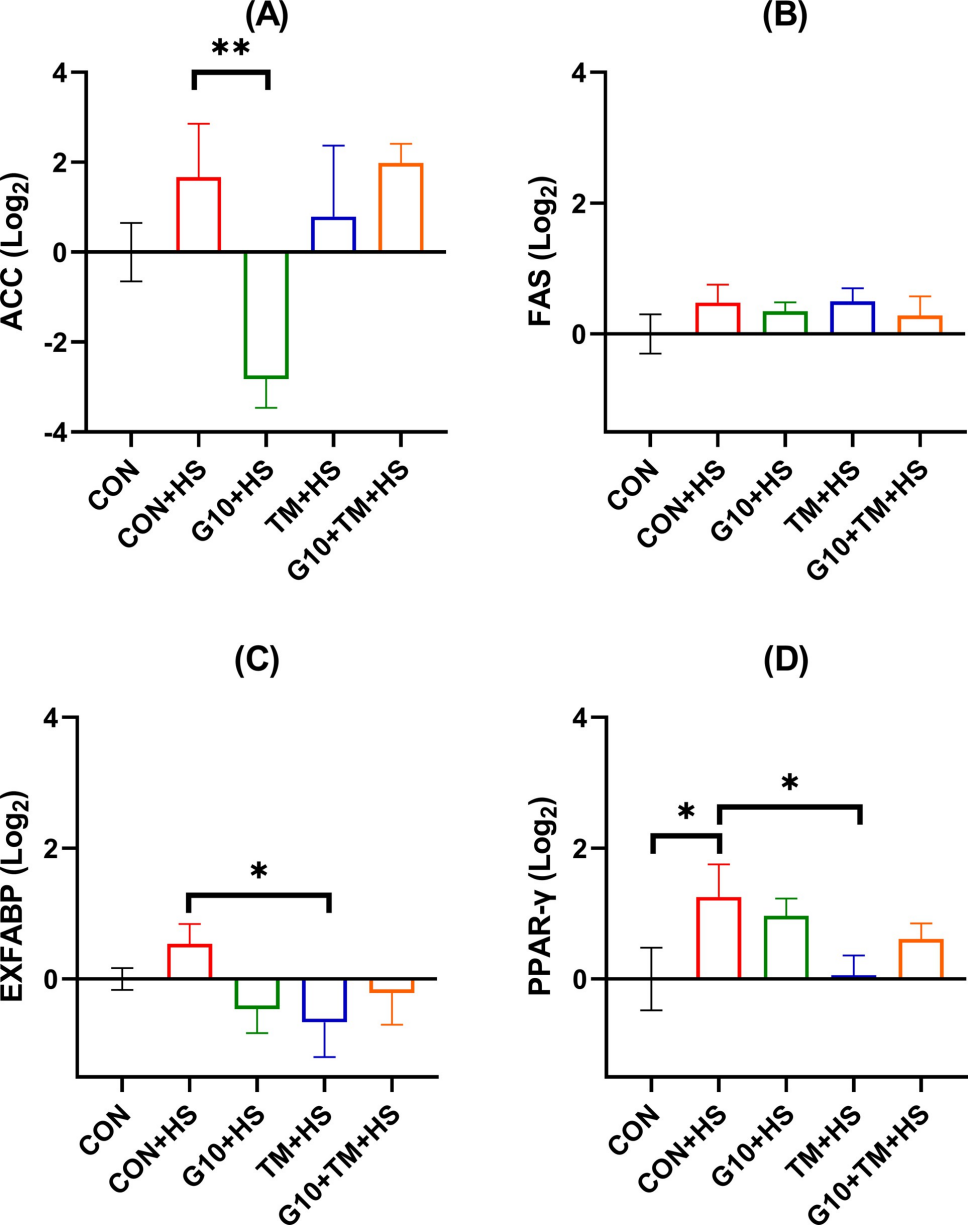
Effects of in ovo feeding of GABA and embryonic thermal manipulation on relative mRNA expression of hepatic ACC (A), FAS (B), EXFABP (C), and PPAR-γ (D) in broiler chickens exposed to cyclic HS. The treatments are described as follows: CON, chicks hatched from control eggs without in ovo injection and incubated at standard temperature; CON+HS, chicks hatched from control eggs without in ovo injection, incubated at standard temperature but exposed to HS; G10+HS, chicks hatched from eggs injected at 17.5 days of incubation with 0.6mL of 10% GABA dissolved in distilled water and exposed to HS; TM+HS, chicks hatched from thermally manipulated eggs exposed to 39.6°C for 6 h daily from ED 10 to 18 and exposed to HS; G10+TM+HS, chicks hatched from eggs that received both previous treatments during incubation and exposed to HS. Data show mean ± SEM (n = 5). Results of the contrast analysis are indicated in the graph. Abbreviations: ACC, acetyl-CoA carboxylase; EXFABP: Extracellular fatty acid-binding protein; FAS, fatty acid synthase; PPAR-γ: Peroxisome proliferator-activated receptor-gamma.

Fig 3 presents the relative mRNA expression of ACC, FAS, EXFABP, and PPAR-γ. GABA or TM did not significantly affect FAS gene expression. When compared to the CON+HS group, the G10+HS and the TM+HS treatments downregulated the expressions of ACC (P = 0.002) and EXFABP genes (P = 0.045), respectively. PPAR-γ gene was increased by HS (P = 0.029) but decreased by the TM+HS (P = 0.045), compared to the CON+HS group. There was no difference (P = 0.358, MANOVA) between treatments when comparing total fatty acid-related gene expression.

### Effects of in ovo feeding of GABA and TM on hepatic heat shock proteins gene expression

**Fig 4:**
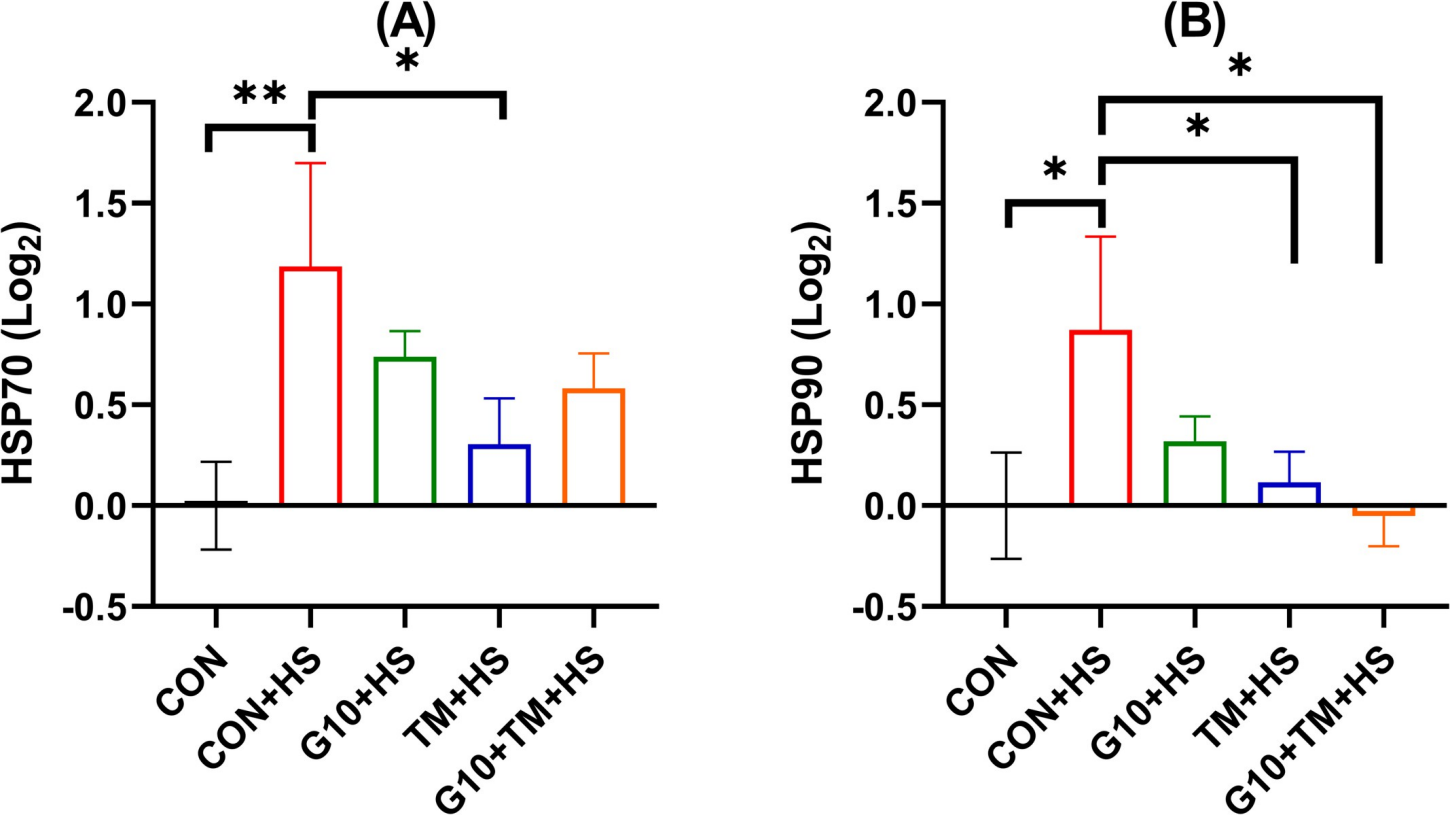
Effects of in ovo feeding of GABA and embryonic thermal manipulation on relative mRNA expression of hepatic HSP70 (A), and HSP90 (B) in broiler chickens exposed to cyclic HS. The treatments are described as follows: CON, chicks hatched from control eggs without in ovo injection and incubated at standard temperature; CON+HS, chicks hatched from control eggs without in ovo injection, incubated at standard temperature but exposed to HS; G10+HS, chicks hatched from eggs injected at 17.5 days of incubation with 0.6mL of 10% GABA dissolved in distilled water and exposed to HS; TM+HS, chicks hatched from thermally manipulated eggs exposed to 39.6°C for 6 h daily from ED 10 to 18 and exposed to HS; G10+TM+HS, chicks hatched from eggs that received both previous treatments during incubation and exposed to HS. Data show mean ± SEM (n = 5). Results of the contrast analysis are indicated in the graph. Abbreviations: HSP70, heat-shock protein 70; HSP90, heat shock protein 90.

**Fig 5:**
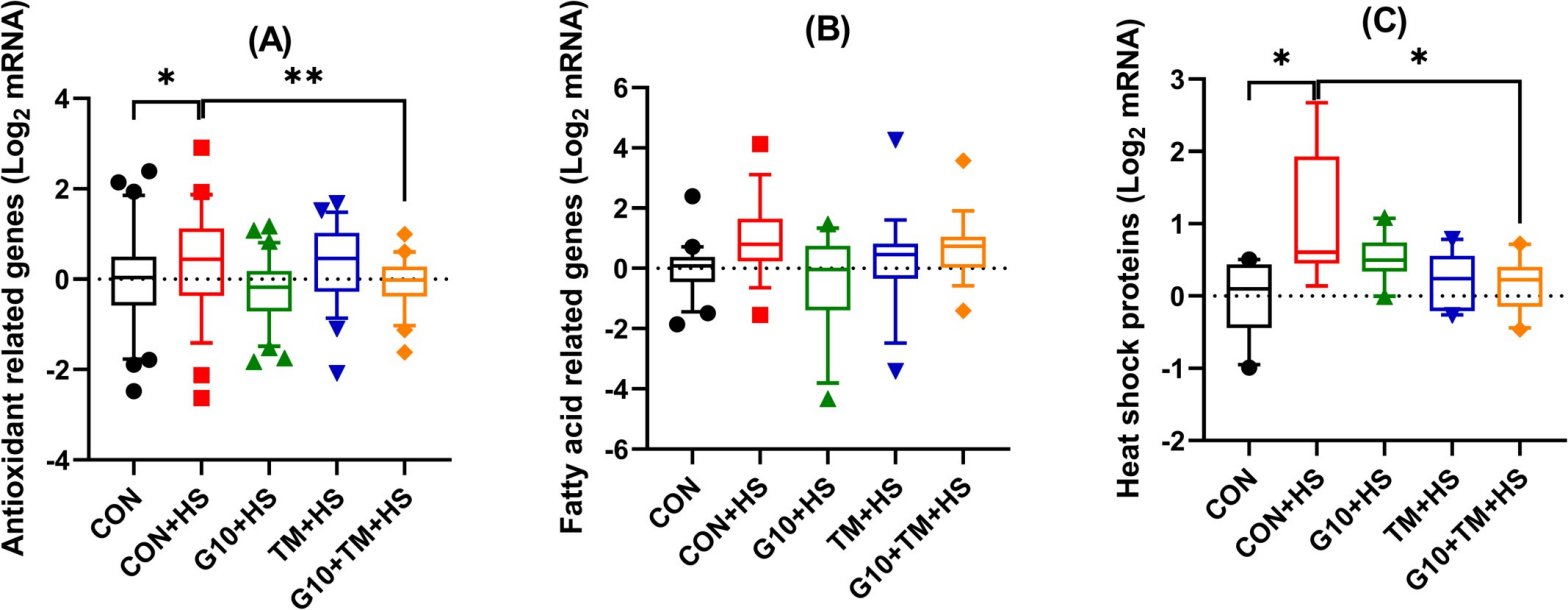
Boxplots showing the assessment of the effects of in ovo feeding of GABA and embryonic thermal manipulation on relative mRNA expression of the set of antioxidant-related genes (A), fatty acid-related genes (B), and heat shock proteins (C) using MANOVA. The treatments are described as follows: CON, chicks hatched from control eggs without in ovo injection and incubated at standard temperature; CON+HS, chicks hatched from control eggs without in ovo injection, incubated at standard temperature but exposed to HS; G10+HS, chicks hatched from eggs injected at 17.5 days of incubation with 0.6mL of 10% GABA dissolved in distilled water and exposed to HS; TM+HS, chicks hatched from thermally manipulated eggs exposed to 39.6°C for 6 h daily from ED 10 to 18 and exposed to HS; G10+TM+HS, chicks hatched from eggs that received both previous treatments during incubation and exposed to HS.

Fig 4 shows the gene expression of HSP70 and HSP90 after in ovo feeding of GABA and TM. The CON+HS group upregulated HSP70 (P = 0.005) and HSP90 (P = 0.022) hepatic gene expression compared to the CON group. Compared to the CON+HS group, the TM+HS group significantly reduced HSP70 mRNA expression (P = 0.031), but the TM+HS and the G10+TM+HS also downregulated (P = 0.043 and P = 0.016) the HSP90 gene. Similarly, there was an overall increase (P = 0.035, MANOVA) of the HSP genes by the CON+HS relative to the CON group (Fig 5C). However, only the G10+TM+HS group significantly decreased (P = 0.042, MANOVA) the overall HSP response compared to the CON+HS treatment (Table 4).

**Table 4:** Results of MANOVA and multivariate planned contrasts on sets of hepatic genes expression in broiler chickens exposed to HS.

Set of genes	MANOVA	Multivariate planned contrast			
		CON vs CON+HS	CON+HS vs G10+HS	CON+HS vs TM+HS	CON+HS vs CON+TM+HS
Fatty acid-related genes	ns	ns	ns	ns	ns
Antioxidant-related genes	0.034	0.020	0.093	ns	0.003
Heat shock proteins	0.035	0.023	ns	0.098	0.042

The treatments are described as follows: CON, chicks hatched from control eggs without in ovo injection and incubated at standard temperature; CON+HS, chicks hatched from control eggs without in ovo injection, incubated at standard temperature but exposed to HS; G10+HS, chicks hatched from eggs injected at 17.5 days of incubation with 0.6mL of 10% GABA dissolved in distilled water and exposed to HS; TM+HS, chicks hatched from thermally manipulated eggs exposed to 39.6°C for 6 h daily from ED 10 to 18 and exposed to HS; G10+TM+HS, chicks hatched from eggs that received both previous treatments during incubation and exposed to HS. Data show mean ± SEM (n = 5). The Wilks’ Lambda P-values are reported. Abbreviation: ns, not significant.

### Association between hepatic gene expression, blood biochemical parameters, and relative liver index

Pearson correlation coefficients between hepatic gene expression and the plasma biochemical parameters revealed that total protein levels in plasma had a significant positive correlation with HSP70 gene expression (r = 0.45, P = 0.028).

**Fig 6:**
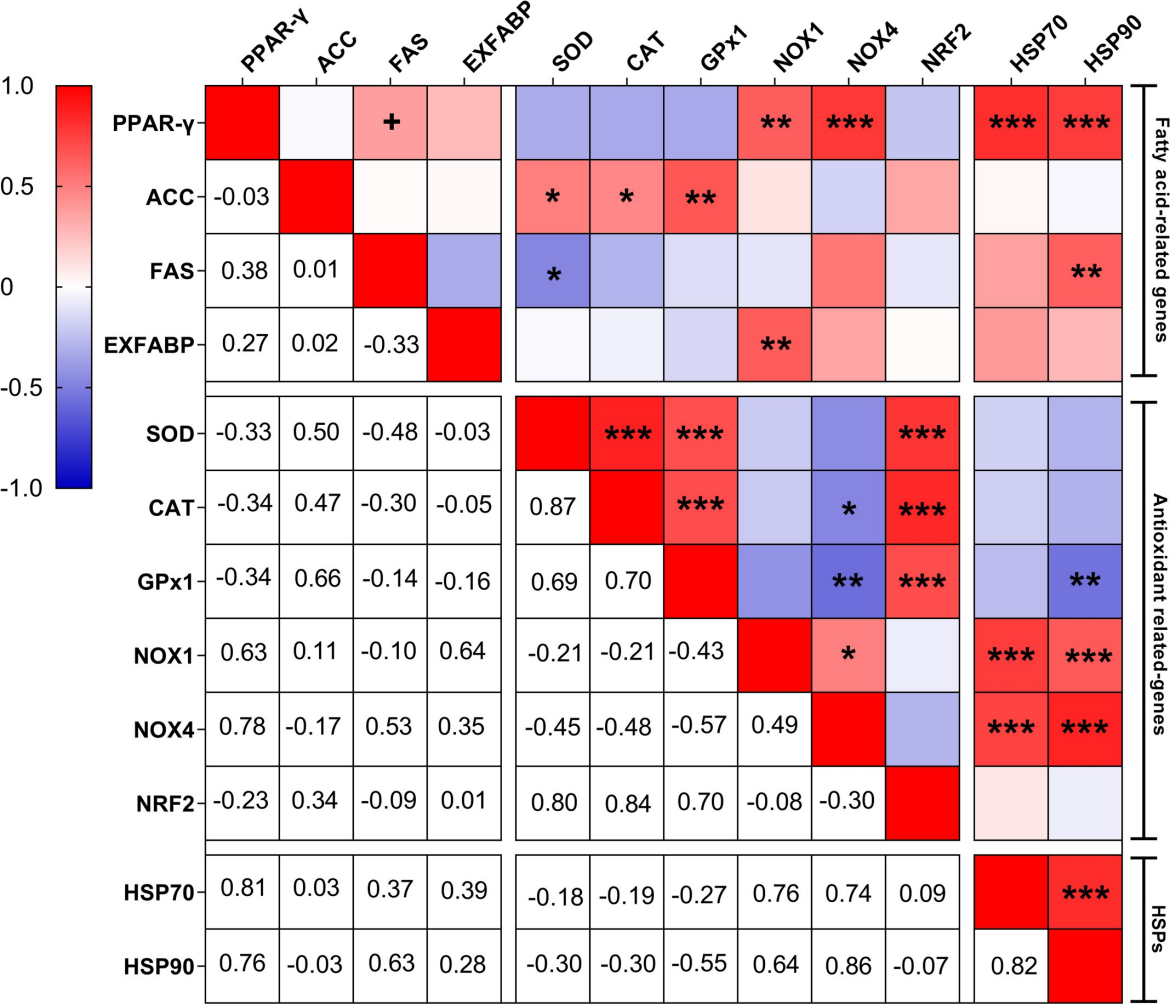
Pearson correlation heat map between the relative mRNA levels of the genes studied. The red color indicates a positive correlation, the blue color indicates a negative correlation and the white color indicates no correlation. Pearson r values were calculated using the “CORR procedure” of the SAS software version 9.4 (SAS Institute Inc., 2009). Abbreviations: ACC, acetyl-CoA carboxylase; CAT, catalase; EXFABP, extracellular fatty acid-binding protein; FAS, fatty acid synthase; GPx1, HSP70, heat-shock protein 70; HSP90, heat shock protein 90; glutathione peroxidase 1; NOX1, nicotinamide adenine dinucleotide phosphate oxidase 1; NOX4, nicotinamide adenine dinucleotide phosphate oxidase 4; NRF2, nuclear factor erythroid 2-related factor 2; PPAR-γ, peroxisome proliferator-activated receptor-gamma; SOD, superoxide dismutase. + Correlation is significant at the P < 0.1. * Correlation is significant at the 0.05 level. ** Correlation is significant at the 0.01 level. *** Correlation is significant at the 0.001 level.

At the same time, associations between gene sets showed interesting results (Fig 6). PPAR-γ had strong positive correlations with HSP90 (r = 0.76, P = 0.001), HSP70 (r = 0.81, P = 0.001), NOX4 (r = 0.78, P = 0.001), and NOX1 (r = 0.63, P = 0.01). ACC had also positive correlations with SOD (r = 0.50, P = 0.014), CAT (r = 0.47, P = 0.028), and GPX1 (r = 0.66, P = 0.002). FAS was negatively correlated with SOD (r = -0.48, P = 0.018) but positively with HSP90 (r = 0.63, P = 0.001). EXFABP and NOX1 were positively correlated (r = 0.64, P = 0.002). Finally, strong positive correlations were observed between the HSPs and NOXs family genes: HSP70 with NOX1 (r = 0.76, P = 0.001); HSP70 with NOX4 (r = 0.74, P = 0.001); HSP90 with NOX1 (r = 0.64, P = 0.001); and HSP90 with NOX4 (r = 0.86, P = 0.001).

## Discussion

The purpose of this study was to evaluate how in ovo feeding of GABA and TM could modulate the hepatic genes expression of broilers exposed to cyclic HS. In ovo feeding and TM have been used separately to improve broilers’ response to HS. Even though the exact mechanism by which in ovo feeding of GABA can provide long-lasting HS tolerance is not yet known, others have shown similar results in the HS response of broilers after in ovo feeding of amino acids such as L-leucine [34–37]. In ovo feeding appears to afford thermotolerance in broiler chicks up to market age. Mostly, in ovo L-leucine injection resulted in lower heat production, higher heat loss, or both in chicks exposed to HS [34]. As metabolic rate is often correlated with body temperature [38], the lower rectal temperature observed after in ovo feeding of L-leucine was indicative of a reduced metabolic rate acquired at later stages of embryonic development. Interestingly, GABA concentration was increased in the brain of the birds hatched after in ovo L-leucine administration [34]. Therefore, both GABA and L-leucine when fed in ovo may be interrelated in the central nervous system such as areas regulating body temperature [39]. Further studies should evaluate how in ovo feeding of GABA might influence body temperature dynamics in broilers.

On the other side, TM has been also the subject of numerous studies. In fact, TM has been initially introduced in the early 2000s as a potential solution to mitigate HS in poultry [40, 41]. The programming effect of TM was thought to be caused by epigenetic regulations leading to long-lasting memory mechanisms [41]. TM also reduced plasma triiodothyronine at hatching and in post-hatched chicks, even under HS [42]. Overall, the current evidence suggests different mechanisms by which TM and in ovo feeding modulate thermotolerance. In this sense, the current study was performed.

Organ weights are important parameters giving insight into the development status of organs. Although all treatment groups did not differ significantly from CON in relative bursa weight, significant differences were detected between treatment groups. The CON+HS and TM+HS groups were significantly higher than the G10+HS and G10+TM+HS, respectively. The results indicate that GABA, but not TM, reversed the increased relative bursa weight induced by HS to normal. The avian bursa of Fabricius is an oval-shaped gland located just above the cloaca and is one of the primary lymphoid organs that produce immunologically competent cells and antibodies [43]. Thus, these results also suggest that the bursa may need to enlarge in response to HS to compensate for the impaired immune function of the birds caused by HS.

In the current study, cyclic HS exposure resulted in a striking augmentation of the relative liver weight. More precisely, the CON+HS group had 62.5% higher relative liver weight compared to the CON group. On the other hand, a lesser increase (16.7 to 20%) was observed in all three treatment groups (G10+HS, TM+HS, and G10+TM+HS). In poultry, the liver is a key organ responsible for primordial functions such as detoxification, removal of waste products, and lipid metabolism [44]. A broiler study highlighted that chronic HS exposure for 14 days led to enlarged liver and higher relative liver weight [45]. Likewise, chronic HS not only increased liver weight, but the difference between heat-stressed and control birds was greater with longer exposure periods (7 vs 14 days) [46]. Interestingly, higher liver weights were consistently associated with hepatic lipid accumulation in both studies. In our study, the lower relative liver weight observed in all treatment groups may suggest reduced lipid accumulation during HS. Therefore, we evaluated some of the major genes involved in fatty acid metabolism in the broilers’ liver.

In ovo feeding of GABA significantly downregulated the expression of ACC in the present study. ACC and FAS are the two key enzymes responsible for fat synthesis in the liver. Both enzymes act together in the sense that ACC catalyzes the synthesis of acetyl-CoA into malonyl-CoA, whereas FAS controls the rate of fatty acid synthesis in tissues [47]. We have previously reported similar results [27]. In addition, in ovo feeding of GABA could reduce ACC gene expression in broilers exposed to four days of cyclic HS [22]. Moreover, dietary GABA could significantly alter ACC gene expression in broilers exposed to chronic HS [48]. Similar findings between the current and previous studies suggest that GABA can effectively limit the first step of lipid accumulation during HS. In chickens, EXFABP was identified as an extracellular protein that ensures the extracellular transport of long-chain fatty acids [49]. Similarly, PPAR-γ was shown to regulate adipogenesis, hepatic lipids synthesis, and triglycerides storage in the liver [50]. Thus, higher expression of these genes in the CON+HS group may be another indicator of lipid accumulation during HS. In the current study, EXFABP and PPAR-γ expressions were both significantly reduced in the birds that received TM, specifying a reduction in lipid accumulation. On the contrary, the impacts of TM on duck embryos show opposite conclusions. TM resulted in higher lipid deposition, whether performed from EDs 11 to 24 with a 1°C higher incubation temperature [51] or from EDs 13 to 27 with a 1.5°C increase temperature [52]. These results not only indicate a species-dependent effect of TM but also suggest other parameters, such as TM severity and duration, to be considered in interpreting the study results. Preferably, further studies should consider the role of TM in lipid metabolism in broilers.

One of the major drawbacks of high rearing temperature in poultry is to increase oxidative stress [5]. Indeed, HS stimulates excessive levels of ROS associated with the disturbance of redox homeostasis [53]. Fortunately, under stressful conditions, the activation of transcription factors such as NRF2 engages the synthesis of three major antioxidant enzymes GPX, SOD, CAT as a palliative measure [54]. Oxidative stress also increases the activity of NOX enzymes which are responsible for the transfer of electrons to oxygen molecules from NADPH, resulting in O_2_^-^ production [55]. In the current study, HS upregulated the expression of NOX4, but TM significantly reduced NOX1 and NOX4 gene expression under cyclic HS. Others also found that TM can lower NOX4 mRNA expression during acute HS [17]. In addition, TM appeared to reduce splenic and hepatic NOX4 gene expression in broilers exposed to chronic HS [56]. Therefore, the reduced expression of NOXs family genes in the TM+HS group observed during heat challenge may contribute to the protection against heat-induced oxidative stress. Even though no significant differences were found in the antioxidant-related genes in the G10+HS group, the G10+TM+HS had the lowest NOX4 gene expression. Moreover, multivariate analysis of antioxidant-related genes suggests that the combination of the two techniques reduced oxidative stress compared to control birds exposed to HS. Taken together, TM and in ovo GABA feeding may synergistically enhance the hepatic antioxidant status of broilers reared at high environmental temperatures.

HSPs are essential biomarkers specifying the levels of cellular integrity against thermal stressors [57, 58]. HSPs can be classified into several categories based on their molecular weights and functions. In avian species, HSP70 and HSP90 are mainly correlated with the development of thermotolerance [59]. In the current study, a significant increase in HSP70 expression was observed concurrently with the upregulation of HSP90 following cyclic HS. As HS induces protein degradation [60] and cell apoptosis [61], cyclic HS can also increase HSPs’ expression. This is the case in the current study. HS increased HSP70 and 90 gene expression, whereas TM reduced these increases, consistent with other’s findings [62]. These results validate the hypothesis that TM improves post-hatch heat tolerance of broilers. The putative mechanism of action by which TM confers heat resistance might suggest a long-lasting memory [63]. Indeed, embryos exposed to high temperatures during their development would later activate thermoregulatory memories acquired when exposed to the same stressor. We particularly observed that when comparing the CON+HS and the G10+TM+HS group, HSP90, but not HSP70, was significantly reduced whereas both were reduced in the TM+HS group. We hypothesized that there may be some additive effect in the G10+TM+HS treatment between in ovo GABA feeding and TM. Therefore, the relatively higher HSP70 expression observed in the G10+TM group might explain the observed outcome. Also, the significant reduction in overall HSP expression in the G10+TM+HS group revealed by multivariate analysis could suggest that the combination of both techniques is likely to be more effective than each taken individually.

The correlation matrix based on the gene expression results revealed interrelation between gene sets. For example, the HSPs and NOXs genes were shown to be positively correlated. HS has been shown to simultaneously increase HSPs and NOXs gene expression [64, 65]. In vitro study also found that the production of HSPs in thermotolerant cells was correlated with NOX activity [66]. Indeed, increased HSPs during HS may preserve the potential of oxidase components to assemble by preventing alterations of the cell’s cytoskeleton structure [67]. Moreover, the positive correlation observed in the present study indicates that the treatments exhibiting lower expression of HSPs also reduced NOX gene expression. Therefore, the current results suggest that thermotolerance acquisition may be associated with reduced ROS generation during HS.

In the current study, no effects of HS were found regardless of the treatment groups. Similarly, a study reported no significant differences in plasma glucose, cholesterol, and triglyceride concentrations in broilers exposed to cyclic HS [68]. On the other side, acute HS resulted in elevated glucose, cholesterol and reduced total protein levels in the plasma of broilers [69]. To mimic daily temperature variations, periods of high ambient temperature and normal breeding temperatures alternated during cyclic HS studies, thus increasing the coping ability of broilers over time [15]. To determine blood parameters, blood samples may be taken immediately after the end of the last cyclic HS period or even the next day. In the current study, samples were taken 24h after the last cyclic HS exposure. Therefore, factors such as HS type and sampling time can influence the inconsistency of results observed in different studies.

Together, the current results show that under HS, in ovo feeding of GABA and TM during incubation can reduce liver weights and modulate ACC, NOX1, NOX4, PPAR-γ, EXFABP, and HSP70 independently. They also indicate that the combination of both techniques results in overall better antioxidant and lower HSP responses to HS. However, further studies including data on growth performance and thermoregulation traits would be necessary to confirm the current findings.

## Supporting information

S1 Fig Scatterplot matrix of genes expression used in the Pearson correlation. The treatments are described as follows: CON, chicks hatched from control eggs without in ovo injection and incubated at standard temperature; CON+HS, chicks hatched from control eggs without in ovo injection, incubated at standard temperature but exposed to HS; G10+HS, chicks hatched from eggs injected at 17.5 day of incubation with 0.6mL of 10% GABA dissolved in distilled water and exposed to HS; TM+HS, chicks hatched from thermally manipulated eggs exposed to 39.6°C for 6 h daily from ED 10 to 18 and exposed to HS; G10+TM+HS, chicks hatched from eggs that received both previous treatments during incubation and exposed to HS. Abbreviations: ACC, acetyl-CoA carboxylase; CAT, catalase; EXFABP, extracellular fatty acid-binding protein; FAS, fatty acid synthase; GPx1, HSP70, heat-shock protein 70; HSP90, heat shock protein 90; glutathione peroxidase 1; NOX1, nicotinamide adenine dinucleotide phosphate oxidase 1; NOX4, nicotinamide adenine dinucleotide phosphate oxidase 4; NRF2, nuclear factor erythroid 2-related factor 2; PPAR-γ, peroxisome proliferator-activated receptor-gamma; SOD, superoxide dismutase.
(TIF)

S1 Table Results of planned contrasts on hepatic genes expression in broiler chickens exposed to heat stress. The treatments are described as follows: CON, chicks hatched from control eggs without in ovo injection and incubated at standard temperature; CON+HS, chicks hatched from control eggs without in ovo injection, incubated at standard temperature but exposed to HS; G10+HS, chicks hatched from eggs injected at 17.5 day of incubation with 0.6mL of 10% GABA dissolved in distilled water and exposed to HS; TM+HS, chicks hatched from thermally manipulated eggs exposed to 39.6°C for 6 h daily from ED 10 to 18 and exposed to HS; G10+TM+HS, chicks hatched from eggs that received both previous treatments during incubation and exposed to HS. Abbreviations: ACC, acetyl-CoA carboxylase; CAT, catalase; EXFABP, extracellular fatty acid-binding protein; FAS, fatty acid synthase; GPx1, HSP70, heat-shock protein 70; HSP90, heat shock protein 90; glutathione peroxidase 1; NOX1, nicotinamide adenine dinucleotide phosphate oxidase 1; NOX4, nicotinamide adenine dinucleotide phosphate oxidase 4; NRF2, nuclear factor erythroid 2-related factor 2; PPAR-γ, peroxisome proliferator-activated receptor-gamma; SOD, superoxide dismutase.
(DOCX)

S1 File Raw data used for statistical analysis in the manuscript. The treatments are described as follows: CON, chicks hatched from control eggs without in ovo injection and incubated at standard temperature; CON+HS, chicks hatched from control eggs without in ovo injection, incubated at standard temperature but exposed to HS; G10+HS, chicks hatched from eggs injected at 17.5 day of incubation with 0.6mL of 10% GABA dissolved in distilled water and exposed to HS; TM+HS, chicks hatched from thermally manipulated eggs exposed to 39.6°C for 6 h daily from ED 10 to 18 and exposed to HS; G10+TM+HS, chicks hatched from eggs that received both previous treatments during incubation and exposed to HS.
(XLSX)
