# Supplementary figures and images for: Embryonic manipulations modulate differential expressions of heat shock protein, fatty acid metabolism, and antioxidant-related genes in the liver of heat-stressed broilers
Source: PLoS One. 2022 Jul 15;17(7):e0269748. doi: 10.1371/journal.pone.0269748 (PMC9286270; doi:10.1371/journal.pone.0269748)

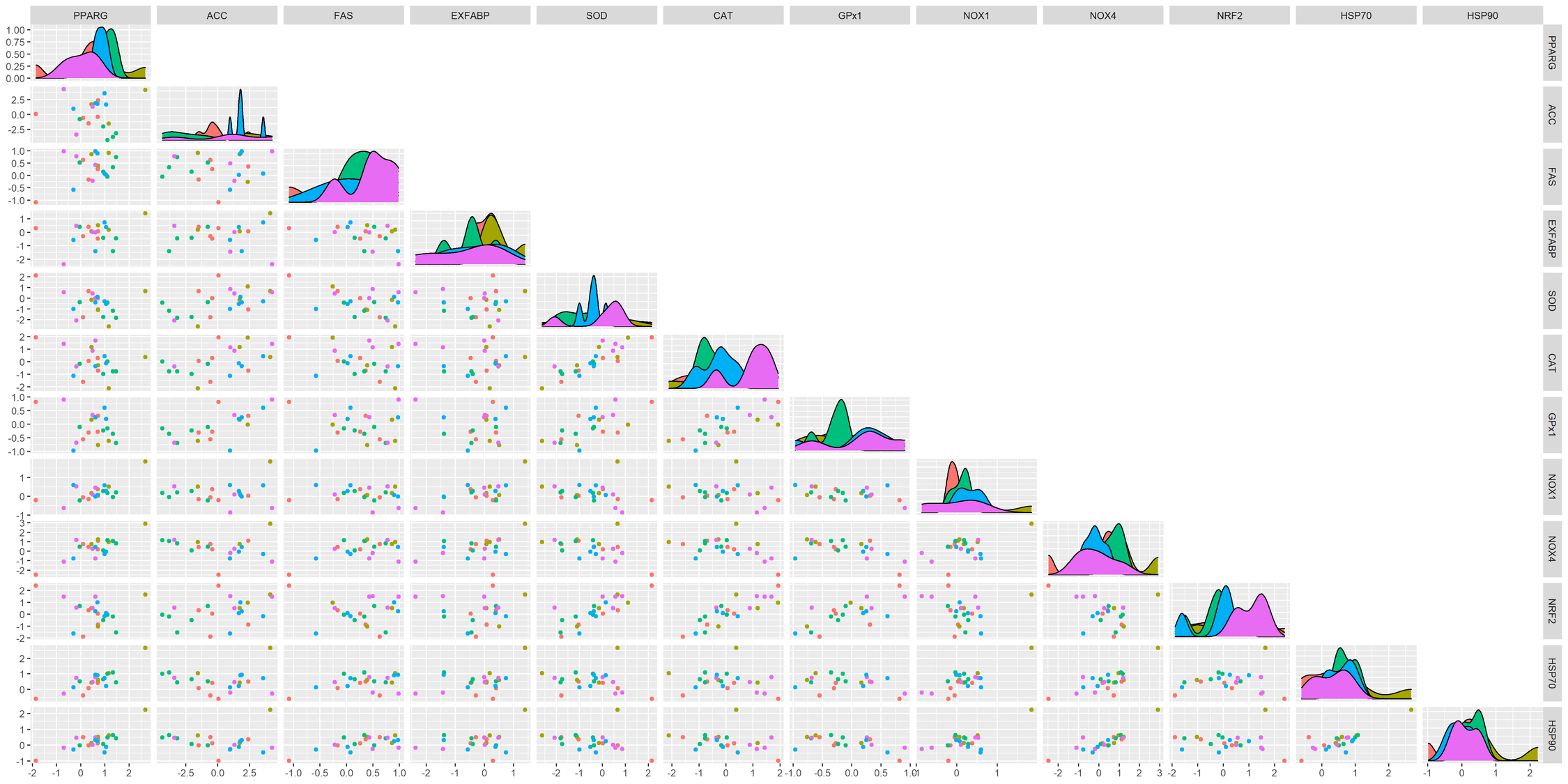

Supplement: S1 Fig — The treatments are described as follows: CON, chicks hatched from control eggs without in ovo injection and incubated at standard temperature; CON+HS, chicks hatched from control eggs without in ovo injection, incubated at standard temperature but exposed to HS; G10+HS, chicks hatched from eggs injected at 17.5 day of incubation with 0.6mL of 10% GABA dissolved in distilled water and exposed to HS; TM+HS, chicks hatched from thermally manipulated eggs exposed to 39.6°C for 6 h daily from ED 10 to 18 and exposed to HS; G10+TM+HS, chicks hatched from eggs that received both previous treatments during incubation and exposed to HS. Abbreviations: ACC, acetyl-CoA carboxylase; CAT, catalase; EXFABP, extracellular fatty acid-binding protein; FAS, fatty acid synthase; GPx1, HSP70, heat-shock protein 70; HSP90, heat shock protein 90; glutathione peroxidase 1; NOX1, nicotinamide adenine dinucleotide phosphate oxidase 1; NOX4, nicotinamide adenine dinucleotide phosphate oxidase 4; NRF2, nuclear factor erythroid 2-related factor 2; PPAR-γ, peroxisome proliferator-activated receptor-gamma; SOD, superoxide dismutase. (TIF) [file pone.0269748.s001.tif]
