# Supplementary material for: Embryonic manipulations modulate differential expressions of heat shock protein, fatty acid metabolism, and antioxidant-related genes in the liver of heat-stressed broilers
Source: PLoS One. 2022 Jul 15;17(7):e0269748. doi: 10.1371/journal.pone.0269748 (PMC9286270; doi:10.1371/journal.pone.0269748)
Supplement: S1 Table — The treatments are described as follows: CON, chicks hatched from control eggs without in ovo injection and incubated at standard temperature; CON+HS, chicks hatched from control eggs without in ovo injection, incubated at standard temperature but exposed to HS; G10+HS, chicks hatched from eggs injected at 17.5 day of incubation with 0.6mL of 10% GABA dissolved in distilled water and exposed to HS; TM+HS, chicks hatched from thermally manipulated eggs exposed to 39.6°C for 6 h daily from ED 10 to 18 and exposed to HS; G10+TM+HS, chicks hatched from eggs that received both previous treatments during incubation and exposed to HS. Abbreviations: ACC, acetyl-CoA carboxylase; CAT, catalase; EXFABP, extracellular fatty acid-binding protein; FAS, fatty acid synthase; GPx1, HSP70, heat-shock protein 70; HSP90, heat shock protein 90; glutathione peroxidase 1; NOX1, nicotinamide adenine dinucleotide phosphate oxidase 1; NOX4, nicotinamide adenine dinucleotide phosphate oxidase 4; NRF2, nuclear factor erythroid 2-related factor 2; PPAR-γ, peroxisome proliferator-activated receptor-gamma; SOD, superoxide dismutase. (DOCX) [file pone.0269748.s002.docx]

Embryonic manipulations modulate differential expressions of heat shock protein, fatty acid metabolism, and antioxidant-related genes in the liver of heat-stressed broilers

Chris Major Ncho^1,2,¶^, Akshat Goel ^1,3,¶^, Vaishali Gupta^2^, Chae-Mi Jeong^1,2^, and Yang-Ho Choi^1,2,3^*

^1^ Department of Animal Science, Gyeongsang National University, Jinju 52828, Republic of Korea;

^2^ Division of Applied Life Sciences (BK21 Plus Program), Gyeongsang National University, Jinju 52828, Republic of Korea;

^3^ Institute of Agriculture and Life Sciences, Gyeongsang National University, Jinju 52828, Republic of Korea;

*Corresponding author

E-mail: yhchoi@gnu.ac.kr;

^¶^ These authors contributed equally to this work.

S1 Table. Results of planned contrasts on hepatic genes expression in broiler chickens exposed to heat stress

| Genes | ANOVA | planned contrasts | | | | | | | | | | | |
| --- | --- | --- | --- | --- | --- | --- | --- | --- | --- | --- | --- | --- | --- |
|  |  | CON vs CON+HS | | | CON+HS vs G10+HS | | | CON+HS vs TM+HS | | | CON+HS vs G10+TM+HS | | |
|  | P-value | Est | SE | P-value | Est | SE | P-value | Est | SE | P-value | Est | SE | P-value |
| PPAR-γ | 0.118 | -0.57 | 0.32 | 0.088 | -0.38 | 0.32 | 0.243 | 0.52 | 0.34 | 0.151 | -0.03 | 0.32 | 0.918 |
| ACC | 0.008 | -0.32 | 0.78 | 0.684 | 3.14 | 0.78 | 0.002 | -0.46 | 0.85 | 0.588 | -1.65 | 0.78 | 0.058 |
| FAS | 0.631 | -0.31 | 0.21 | 0.162 | -0.02 | 0.21 | 0.912 | -0.17 | 0.21 | 0.435 | 0.039 | 0.21 | 0.858 |
| EXFABP | 0.293 | 0.16 | 0.33 | 0.641 | 0.3 | 0.36 | 0.423 | -1.65 | 0.33 | 0.045 | 0.05 | 0.36 | 0.882 |
| SOD | 0.573 | 0.39 | 0.45 | 0.401 | 0.7 | 0.45 | 0.139 | -0.35 | 0.45 | 0.448 | 0.01 | 0.45 | 0.973 |
| CAT | 0.261 | -0.07 | 0.42 | 0.868 | 0.061 | 0.42 | 0.172 | -0.88 | 0.42 | 0.051 | 0.35 | 0.46 | 0.448 |
| GPx1 | 0.559 | 0.07 | 0.21 | 0.722 | 0.22 | 0.21 | 0.305 | -0.28 | 0.23 | 0.244 | -0.09 | 0.23 | 0.677 |
| NOX1 | 0.066 | -0.19 | 0.04 | 0.057 | 0.11 | 0.08 | 0.093 | 0.27 | 0.07 | 0.034 | -0.11 | 0.19 | 0.564 |
| NOX4 | 0.088 | -0.38 | 0.19 | 0.032 | -0.34 | 0.38 | 0.371 | 0.5 | 0.19 | 0.021 | 0.55 | 0.21 | 0.024 |
| NRF2 | 0.263 | -0.24 | 0.42 | 0.571 | 0.55 | 0.42 | 0.206 | -0.86 | 0.42 | 0.058 | 0.33 | 0.42 | 0.443 |
| HSP70 | 0.053 | -0.56 | 0.12 | 0.005 | -0.17 | 0.22 | 0.448 | 0.25 | 0.09 | 0.031 | -0.02 | 0.22 | 0.931 |
| HSP90 | 0.105 | -0.25 | 0.08 | 0.022 | -0.06 | 0.21 | 0.748 | 0.13 | 0.05 | 0.043 | 0.31 | 0.12 | 0.016 |

The treatments are described as follows: CON, chicks hatched from control eggs without in ovo injection and incubated at standard temperature; CON+HS, chicks hatched from control eggs without in ovo injection, incubated at standard temperature but exposed to HS; G10+HS, chicks hatched from eggs injected at 17.5 day of incubation with 0.6mL of 10% GABA dissolved in distilled water and exposed to HS; TM+HS, chicks hatched from thermally manipulated eggs exposed to 39.6°C for 6 h daily from ED 10 to 18 and exposed to HS; G10+TM+HS, chicks hatched from eggs that received both previous treatments during incubation and exposed to HS. Abbreviations: ACC, acetyl-CoA carboxylase; CAT, catalase; EXFABP, extracellular fatty acid-binding protein; FAS, fatty acid synthase; GPx1, HSP70, heat-shock protein 70; HSP90, heat shock protein 90; glutathione peroxidase 1; NOX1, nicotinamide adenine dinucleotide phosphate oxidase 1; NOX4, nicotinamide adenine dinucleotide phosphate oxidase 4; NRF2, nuclear factor erythroid 2-related factor 2; PPAR-γ, peroxisome proliferator-activated receptor-gamma; SOD, superoxide dismutase.
